# A case of aspergillus endophthalmitis in an immuncompetent woman: intra-ocular penetration of oral voriconazole: a case report

**DOI:** 10.1186/1757-1626-3-31

**Published:** 2010-01-18

**Authors:** SA Logan, MS Rajan, E Graham, EM Johnson, JL Klein

**Affiliations:** 1 Departments of Infection, St Thomas' Hospital, Lambeth Palace Road, London SE1 7EH, UK; 2 Department of Ophthalmology, St Thomas' Hospital, Lambeth Palace Road, London SE1 7EH, UK; 3 Mycology Reference Laboratory, HPA Southwest Laboratory, Myrtle Road, Kingsdown, Bristol, BS2 8EL UK

## Abstract

**Background:**

There are very few reports of Aspergillus fumigatus causing endogenous endophthalmitis (EAE) in immunocompetent individuals although it is well recognised in the immunocompromised. Treatment can be with intravitreal, intravenous and oral antifungal agents. The benefit of an oral agent is clear however the concentration of voriconazole in the inflamed eye after oral administration has not previously been documented.

**Case presentation:**

We present a case of EAE in an immunocompetent 78-year-old Caucasian female who was subsequently managed with oral voriconazole. Using a bioassay, we show an appropriate voriconazole concentration in serum and vitreous samples.

**Conclusion:**

This case adds to the limited literature on the prevalence of endogenous endophthalmitis in immunocompetent patients and supports the use of voriconazole in such cases.

## Background

Endogenous *Aspergillus *Endophthalmitis (EAE) is a condition most commonly seen in patients who are immunosuppressed. There are only five reports in the literature of the condition in immunocompetent individuals. Loss of visual acuity is a common presentation and patients frequently lose sight in the affected eye, particularly if there is a delay in instituting appropriate therapy. Voriconazole and other new anti-fungal agents are likely to play an increasing role in treating this condition.

## Case presentation

A 78-year-old white caucasian lady presented to her local hospital with rapid loss of vision in her right eye over 12 hours. She had been suffering from non-specific joint pains for several months for which she had been receiving acupuncture. Ten days prior to her admission a rheumatologist had diagnosed polymyalgia rheumatica based on her symptomatology and scalp tenderness, but she had not been started on steroid treatment. She had had an anterior resection for adenocarcinoma of the bowel in 2001 and a computed tomography scan six months previously showed no evidence of tumour recurrence. There was a past medical history of melanoma (resected from her right foot) in 1983, and a transient ischaemic attack but had no history of diabetes, ocular trauma or sinusitis. There had been no recent hospital admissions or medical procedures.

**Figure 1:**
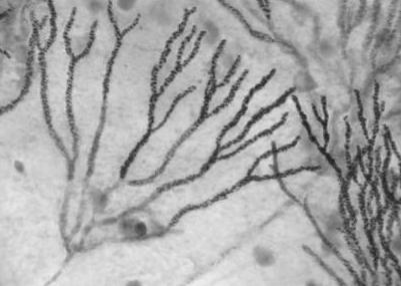
**Gram Stain of first vitreous aspirate**.

Initial examination revealed a reduction in her visual acuity on the right to hand movements only, there was also tenderness over the right temporal artery. The left eye vision was normal. The right fundus seen with a direct ophthalmoscope showed an elevated, yellow, sub retinal macular lesion with associated retinal haemorrhages and cotton wool spots. There was no evidence of uveitis. The physical examination was otherwise normal. At this time her erythrocyte sedimentation rate was 58 mm/hr, her C reactive protein 1 mg/L, total white cell count of 11.9 × 10^9^/L. Giant cell arteritis was suspected to be the cause of her pain and she was commenced on 80 mg of prednisolone daily. After five days the pain worsened and became more localised to the right eye and she developed panuveitis with 1 mm hypopyon in the anterior chamber. She was transferred to our institute for further management. An ultrasound B scan of the eye showed retinal elevation at the macula, choroidal thickening and subretinal fluid suggesting the diagnosis of endogenous endophthalmitis. A vitreous biopsy was performed which showed the macular lesion to orginate from a subretinal mass accompanied by an area of macular retinitis with intense inflammatory exudate in the posterior vitreous. Intravitreal ceftazidime and vancomycin were administered. The Gram stain of the vitreous biopsy showed moderate numbers of neutrophils, branching septate hyphae and no bacteria (Figure 1). Bacterial cultures were negative but culture on Sabouraud's agar revealed a mould identified as *Aspergillus fumigatus *on the basis of typical colonial and microscopic morphology. The organism was susceptible to voriconazole (MIC 0.25 μg/ml) using the Clinical and Laboratory Standards Institute MS27-A2 method[1]. She was commenced on oral voriconazole 400 mg twice a day for 24 hours as a loading dose. The following day she complained of visual hallucinations at night, a well-known side effect of the drug and the agent was discontinued for a day. She subsequently tolerated maintenance treatment at a dose of 200 mg 12 hourly to complete a two-week course. Systemic steroids were tapered and discontinued over two weeks. Six days after commencing voriconazole she underwent a second vitreous biopsy with intravitreal injection of 5 mcg of amphotericin B. Gram stain and culture of this sample were negative. Voriconazole levels were measured in serum and vitreous aspirate, both eight hours after her last 200 mg dose, by means of a bioassay. Both the assay of the serum and the vitreous revealed a concentration of 0.69 μg/ml.

An extensive search for a source of her infection (blood cultures, echocardiogram, computed tomography of chest, abdomen and pelvis and magnetic resonance imaging of the brain) was negative. The uveitis improved substantially during the first week of treatment, but there was no improvement in her visual acuity due to the macular involvement. On review two months later the uveitis had settled, but there was persistent pain and low intraocular pressure (4 mmHg) in the right eye indicating the onset of phthisis bulbi. She was blind in the right eye. Therefore enucleation of the right eye was suggested and the patient consented to the procedure. Histological examination of the enucleated eye showed non-specific chronic fibrosing vitritis with no fungal elements seen using special stains.

## Discussion

A review of the literature revealed only five other cases of endogenous *Aspergillus *endophthalmitis (EAE) in immunocompetent patients [2-6].

Recognised risk factors include a history of immunocompromise, malignancy, organ transplantation, long term corticosteroid use, drug abuse, ocular surgery, trauma, endocarditis and chronic obstructive airways disease [3,5-8].

A comprehensive search for a portal of entry or a further focus of *Aspergillus *infection was conducted in our case to no avail. Interestingly, as in our patient, three of the reported immunocompetent cases had been treated with corticosteroids for their symptoms prior to a diagnosis being made [3-5]. It would be reasonable to assume that this administration in the absence of an anti fungal agent may have worsened the condition. Poor outcome in the context of EAE is well recognised. In the largest review of EAE [8], the outcomes with intravitreal and intravenous antifungal agents only 8% (n = 84) regained useful visual acuity. The propensity of *Aspergillus *for the macula region in the retina was directly correlated to poor visual outcome in these studies [8,9].

Voriconazole is a second generation synthetic derivative of fluconazole. It has a 96% oral bioavailability and reaches peak plasma concentrations two to three hours after oral dosing [7].

The ocular penetration of voriconazole was examined in 14 patients undergoing elective pars plana vitrectomies[10]. The voriconazole concentrations measured two to three hours after two oral doses of 200 mg were 2.13 +/- 0.93 μg/ml (serum), 0.81 +/- 0.31 μg/ml (vitreous) and 1.13 +/- 0.57 μg/ml (aqueous). Although our levels were measured eight hours after the last dose of voriconazole and a different assay was used, the vitreous concentration was similar to that in the above study and equivalent to the serum level. These results suggest that the ocular penetration of voriconazole may be enhanced in the context of endophthalmitis where there is a break in the blood aqueous and retinal barrier. We postulate this as the reason for enhanced permeability of the drug into the eye to match serum concentrations. The voriconazole concentration measured in our case was above the MIC_90 _of voriconazole for *Aspergillus fumigatus *(0.5 μg/ml). Voriconazole is an attractive agent for treatment of fungal endophthalmitis; it has a broad spectrum of activity, it can be given orally, and although visual side effects are common, they usually resolve after the first week of treatment. Amphotericin B, by contrast, can only be given parenterally and may be toxic when administered intravitreally. Another promising agent is caspofungin possibly in conjunction with voriconazole.

## Conclusion

This report adds to the small number of published cases of EAE in the absence of known risk factors. We have also shown enhanced intraocular penetration of voriconazole in the context of endophthalmitis demonstrating the potential role of the drug in treating infection and limiting the progression of the disease. The poor outcome, however, highlights the problems encountered in treating Endogenous *Aspergillus *Endophthalmitis and emphasises the need for early diagnosis and appropriate institution of systemic antifungal therapy.

## Abbreviations

EAE: Endogenous Aspergillus Endophthalmitis.

## Consent

Written informed consent was obtained from the patient for publication of this case report and accompanying images. A copy of the written consent is available for review by the Editor-in-Chief of this journal.

## Competing interests

The authors declare that they have no competing interests.

## Authors' contributions

SL and JK treated the patient and are the major contributors to this report. MR and EG were part of the patients inpatient team. EJ performed the bioassay. All authors have reviewed and approved the final manuscript.
